# Spiritual Healing in the Treatment of Rheumatoid Arthritis: An Exploratory Single Centre, Parallel-Group, Double-Blind, Three-Arm, Randomised, Sham-Controlled Trial

**DOI:** 10.1155/2014/269431

**Published:** 2014-12-31

**Authors:** Henning Bliddal, Robin Christensen, Lars Højgaard, Else Marie Bartels, Karen Ellegaard, Robert Zachariae, Bente Danneskiold-Samsøe

**Affiliations:** ^1^The Parker Institute, Department of Rheumatology, Copenhagen University Hospital, Bispebjerg and Frederiksberg, Nordre Fasanvej 57, 2000 Copenhagen, Denmark; ^2^Faculty of Health Sciences, University of Southern Denmark, Campusvej 55, 5230 Odense M, Denmark; ^3^Danish Healing Research Center, Stationsvej 16, 3210 Vejby, Denmark; ^4^Unit for Psychooncology and Health Psychology, Department of Oncology, Aarhus University Hospital and Department of Psychology and Behavioral Science, Bartholins Allé 9, 8000 Aarhus C, Denmark

## Abstract

Our objective was to investigate the efficacy of “energy/spiritual healing” in rheumatoid arthritis (RA). Eligible patients were women with RA on stable medication. The design was a randomised, blinded, sham-controlled trial; the third group included an external unblinded control of the natural course of RA. Participants in both groups received 8 sessions with “perceived healing” over 21 weeks with 8 weeks of follow-up. Active healing (AH) treatment comprised healing with no physical contact, and sham healing (SH) included exactly the same healing with a sham healer. During intervention, participants wore hearing protectors and were blindfolded. No healing (NH) only had their outcomes assessed. Coprimary outcomes were disease activity score (DAS) for 28 joints and Doppler ultrasound. All 96 patients randomised were handled as the intention-to-treat population, using a baseline-carried forward approach to replace the missing data. Eighty-two (85%) participants completed the 29-week trial. At end point (week 29), mean difference in DAS28 between AH versus SH was statistically but not clinically significant in favour of AH (0.62 DAS28 points; 95% CI: 0.13 to 1.11; *P* = 0.014), while no differences between groups occurred in Doppler ultrasound. There are no clear physiological or psychological explanations for the findings in this tightly controlled study. The trial data indicates a need for independent replication.

## 1. Introduction

Despite all efforts with medical therapy of rheumatoid arthritis (RA), many patients have some persisting symptoms, which may be one of the reasons why a significant number of patients also add self-prescribed complementary and alternative medicine (CAM) [1] in many cases from the onset of disease and more than 90% of patients will use one or more types of CAM [2]. Whether this is directed towards the arthritis or towards other concomitant ailments is not clear; however, CAM presents a parallel medication throughout the course of the disease [3] with a considerable economic burden for some patients who spend as much or more money on CAM as they do on the conventional prescriptions from their physician [4]. Although the medical community in general has been reluctant to accept CAM [5], keeping an open mind towards CAM will give physicians a more complete insight into their patients' compliance with therapy [2].

Of the many categories of CAM, spiritual healing may be regarded as one of the most intriguing, since this treatment has no medicinal properties and is unlikely to interfere with the conventional medications in any known physiological way. Spiritual healing has been defined as a systematic, purposeful intervention by one or more persons by means of focused intention to improve their condition [6, 7]. Personal belief in a treatment may per se lead to an improvement, and total blinding of the patient and observer to the treatment is necessary in tests of healing. It has been speculated that the therapeutic effect of healing is a result of the “channelling” of a so far unidentified form of energy from an undefined source, via the healer to the patient. The central claim of spiritual healers is that this process facilitates self-healing in the patient. Spiritual healing includes several categories, including “therapeutic touch” and “intercessory prayer,” and the healing may be attributed to God, spirits, universal forces or energies, biological healing energies residing in the healer, or self-healing powers or energies thought to reside latent in the healed organism [8]. The highly controversial hypothesised effects of distant or spiritual healing contradict our traditional sense of reality and are in conflict with what is generally viewed as being in accordance with physiological science.

The objective of the study was to test the hypothesised effect of spiritual healing on RA using disease activity score (DAS) using 28 joints and Doppler ultrasound as the coprimary outcome.

## 2. Methods

### 2.1. Trial Design

The study was designed as an exploratory single centre, randomised (stratified by minimization (1 : 1 : 1)), double-blind, sham- and no attention-controlled, parallel-group, 29-week trial, conducted in Denmark (Clinicaltrials.gov NCT00967395). The design included active healing performed by an experienced professional healer, a sham healing with a stand-in person with no experience of healing, and a control group allocated to no treatment.

### 2.2. Participants

Eligible patients were women 18 years of age or older with RA according to the 1987 revised American College of Rheumatology criteria [9] but who were not selected on the basis of their level of activity. The treatment of both the RA and any other medical condition had been stable and constant for at least three months at the time of enrolment, and no future planned changes of therapy existed at the time of inclusion. Major exclusion criteria were changes in therapy for other medical diseases and changes in physical or other types of therapy for the RA, including CAM. Oral corticosteroids, if used previously, were allowed at a maximum prednisone dose an equivalent of 10 mg/day. Increases in corticosteroid doses were not permitted. No intra-articular corticosteroid injection was allowed between inclusion and end point outcome assessment.

### 2.3. Outcome Measures

All clinical assessments were carried out at baseline, at 21 weeks, and at follow up, 29 weeks from baseline. The coprimary outcomes were the disease activity score 28 based on C-reactive protein (DAS28-CRP) [10, 11] and a quantitative measure of the colour Doppler ultrasound [12] both handled as continuous variables [13]. The DAS28-CRP index combines information relating to the number of swollen and tender joints out of a possible 28 joints, in addition to a measure of general health and the acute phase response (CRP). The DAS28-CRP is a composite measure, with a score ranging from 0 to 9.4, which can be used to objectively evaluate a patient's response to treatment [11]. Doppler ultrasound is as follows: an investigator trained in musculoskeletal ultrasound (KE) performed all examinations according to a protocol, which has been shown to have excellent reliability [14]. Scanning was performed with a Siemens Acuson Sequoiaä (Mountain View, CA, USA) using a 14 MHz centre frequency linear array transducer. The colour Doppler was adjusted for maximum sensitivity for low flow (Nyquist limit 0.014 m/s, lowest wall filter, and 7 MHz Doppler frequency) with Doppler gain just below the noise level [15]. The joint was scanned from the dorsal side in the radial, central, and ulnar positions. All scans were performed in the longitudinal plane. Based on the three scans, the mean colour fraction was computed.

Secondary outcome measures were the components included in the DAS28: CRP is the number of tender and swollen joints, patient's global assessment of disease activity. Before randomisation and at follow-up, participants were asked about their attitudes towards CAM and healing, if they had tried one or both and their expectation concerning the effects. This was done using 0–100 mm visual analogue scales (VAS) with the anchors “0” defined as “no, not at all” or “fully dissatisfied” and “100” as “yes, definitely” or “fully satisfied.”

Assessment of safety is as follows: safety measures were collected at weeks 21 and 29, with participants being asked if they had experienced any side effects and if so, what these were. Safety was assessed through the recording of adverse events, physical examinations, and standard laboratory tests. All joint examinations and physician global evaluations of disease activity were performed by the same experienced rheumatologist (HB), who was blinded to group allocation. At the same time points, the patient completed 0–100 mm VAS for the following questions: How did you feel about participating in this project? Do you think that you were treated by the healer? Do you believe that healing is good for arthritis? Will you use other kinds of complementary treatment? Will you proceed to seek treatment by a healer?

### 2.4. Group Allocation and Intervention

The patients were fully informed about the study design, including the chance of being allocated to sham or no treatment, prior to signing the informed consent. Patients were instructed to report any unforeseen changes in medication or disease status during the study. Patients were randomly assigned to receive, either “active healing” (AH), “sham healing” (SH), or “no healing” (i.e., no additional treatments at all) (NH). The randomization was based on minimization [16], thereby randomly allocating patients to one of three groups according to important predictors of disease prognosis: (i) concomitant biological therapy (yes versus no); (ii) disease duration, short (<3 years) versus long (≥3 years); and (iii) age in years [17]. Using this approach enabled concealed group allocation hidden in an external computer, providing the research team with a new list of “where to go” room assignments each time.

#### 2.4.1. Active Healing

Patients in the AH group received “energy healing” provided by a healer working full time as a professional healer with 20 years of experience. The same healer (LH) conducted all healing sessions, using a technique described by the healer as focusing on “channeling spiritual forces,” with each session lasting for 14 minutes. The proposed way of action from the healer's perspective is that he begins the procedure by being aware of his own “spiritual level of consciousness.” From this level, he focuses on “being the connection between inner spiritual entities and the soul of the patient.” Subsequently, the healer attempts to function as “a transmitter of the healing forces into the personality level of consciousness of the patient.” This gives the soul level of the patient a possibility to “enforce the influence on the blockages between soul and personality.” “Individual blockages” are then addressed, and, if possible, resolved. This description is in concordance with the general definition of “spiritual” or “energy” healing as a systematic, purposeful intervention by one or more persons aiming to help another living being (person, animal, plant, cell, or other living system) by means of focused intention to improve their condition [7]. So, as to eliminate any “nonspecific” psychotherapeutic effects, the healer was hidden from the patient behind a screen and the healing conducted without any verbal or nonverbal communication between the healer and the patient.

#### 2.4.2. Sham Healing

In the sham “nonactive” healing control condition, a medical student with no training or experience with healing entered the room. As in the active healing condition, the sham healer was separated from the patient by a screen with no verbal or nonverbal communication between sham healer and patient.

#### 2.4.3. No Healing

Patients allocated to the no healing group were only seen by the ordinary investigators at baseline and the follow-up control sessions.

### 2.5. Procedure

The outpatient clinic is constructed with two corridors and examination rooms in between with two doors in each, one leading to the front and one to the back, corridor. Patients were admitted through the front corridor, while the back corridor was for staff only. On specific days during the study, members of the project staff were divided into two groups: one group tending the patients in the front corridor and the other group directing the healer and sham-healers participating in treatment in the back corridor. Patients were only admitted through the front door, when the back door was closed. Hearing protectors were put on to exclude any sound stimulation; the patients were blindfolded and placed in the supine position on a couch. A screen divided the room with the couch on the front side of the screen and a chair on the backside. No conversation or any other exchange of stimuli through the screen took place during the session. When the patient had been instructed to lie down and the staff member had left the room through the front door and closed it, the staff members in the back corridor were notified over the intercom. Then, the male healer or the sham healer was given instructions to enter their respective rooms. The patients allocated to either the healing group or the sham group received their treatment in rooms, which were changed on all occasions in a concealed protocolised way. No exchange of information took place between the staff in the front and back corridors.

*Concomitant Therapy*. Any medication being used at the time of randomisation was continued, including DMARDs and/or biologics. Doses and routes of administration of concomitant RA therapies, such as anti-TNFs, MTX, corticosteroids, and nonsteroidal anti-inflammatory drugs (NSAIDs), were kept constant throughout the study.

### 2.6. Statistical Methods

The DAS28 level at 8 weeks of follow-up (week 29) was the primary end point. This exploratory study was designed to be able to detect a large clinical effect (Cohen's *d* = 0.8 [18]) corresponding to, with greater than 80% statistical power at a 2-sided level of significance of 5%, comparing active healing with sham healing a difference between groups for DAS28 of 1.2 units. Anticipating a standard deviation (SD) of 1.5, 26 patients were required in each of the two groups of primary interest (active healing versus sham healing). A drop-out rate of around 10% was expected and it was planned to include 6 strata of 15 patients each, enrolling at least 90 RA patients as the intention-to-treat (ITT) population.

ITT principle was based on the total number of randomised patients, irrespective of why the data were missing, and assuming no change from baseline, data were imputed with the baseline-observation carried forward (BOCF) technique (i.e., nonresponder analysis). Patients who withdrew from the study, or had a significant change in the traditional medical therapy after inclusion, were excluded from the per-protocol population, and thus were handled as the ITT (nonresponder) population. In sensitivity analyses of the coprimary outcomes, slightly different analysis scenarios were applied, reflecting available-case analysis, or multiple imputation techniques. At week 29, the AH, SH, and NH groups were compared by the analysis of covariance (ANCOVA) for mean changes from baseline in DAS28, colour fraction (Doppler ultrasound), and the secondary continuous outcomes. The model included the change as the dependent variable, with treatment group being the main effect and the baseline score being the additional covariate. According to the protocol, two primary treatment comparisons were performed: (1) AH versus NH and (2) AH versus SH.

Unless stated otherwise, results are expressed as the difference between the group means and 95% confidence intervals (95% CIs) with the associated *P* values, based on the ANCOVA model.

## 3. Results

### 3.1. Recruitment and Participant Flow

**Figure 1:**
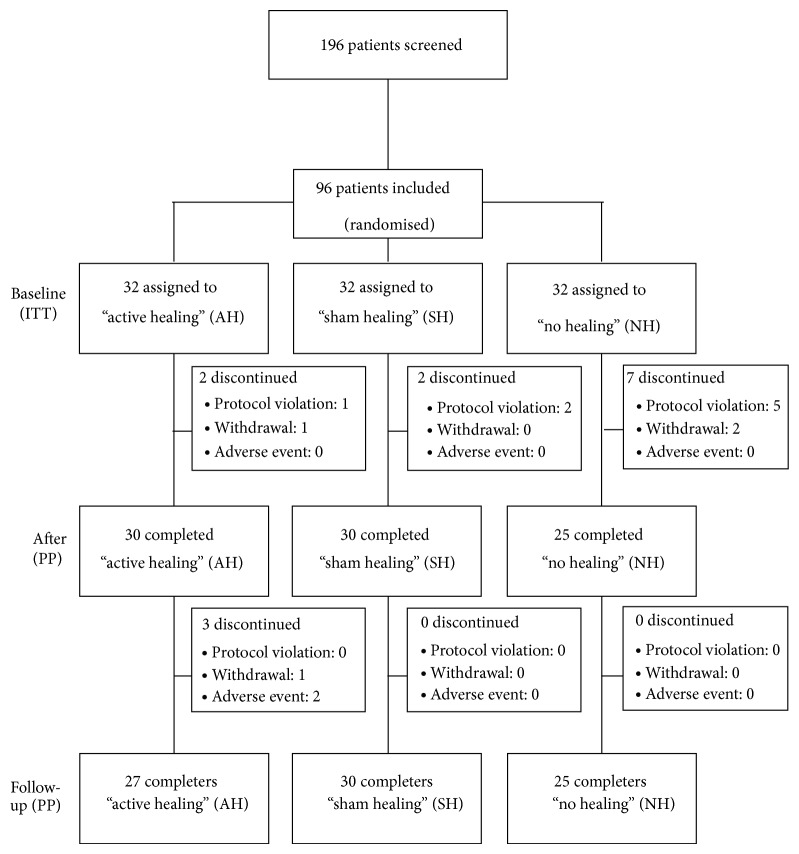
Trial profile. ITT: intention-to-treat. PP: per protocol; the number of patients who did not violate the protocol and remained in the study.

Following the approval by the Frederiksberg and Copenhagen Municipalities' Ethics Committee (KF 01-123/04), patients were recruited at the outpatient clinic at the Department of Rheumatology, Frederiksberg Hospital, Denmark. Data were collected between July 2006 and 31 December 2008, when all patients had completed the first 21 weeks followed by 8 weeks of follow-up. As presented in Figure 1, 196 patients were interviewed by phone using the set criteria to assess eligibility for participation. Of these, 104 were seen in the outpatients' clinic for screening and further information. Ninety-six patients met the criteria, accepted participation, and were enrolled and randomised equally to one of three groups: active healing (*N* = 32), sham healing (*N* = 32), or no healing (*N* = 32). Concomitant use of DMARDs at baseline was prednisolone monotherapy in 4 patients; methotrexate alone or in combination with prednisolone or sulfasalazine in 49, biologics in 14; sulfasalazine or antimalarials as monotherapy in 12, and finally no DMARD therapy in 17 patients. During the 29-week period, 14 of the 96 patients dropped out of the study with no significant difference between groups (Figure 1).

### 3.2. Characteristics of the ITT Population

Table 1 summarizes the baseline demographic and clinical characteristics by treatment group. As expected from the randomisation, there were no statistically significant differences between the groups at baseline. There was no significant difference between groups for any of the variables. The enrolled women were on average 59 years of age, ranging from 27 to 86. The RA in the majority of patients was relatively well treated with a disease status according to DAS28 of 3.68. Some participants had refrained from traditional therapy, as reflected by the range of DAS28 from 1.40 to 7.91 and quartile range from 2.69 to 4.47. The median number of joints assessed as being tender and/or swollen was 3, ranging from 0 to 28, thus indicating the majority of patients being characterised by a low disease status. This finding was supported by the physician's global assessment, showing a median disease activity of 15/100 (range: 0 to 88).

### 3.3. Outcomes and Estimation

**Figure 2:**
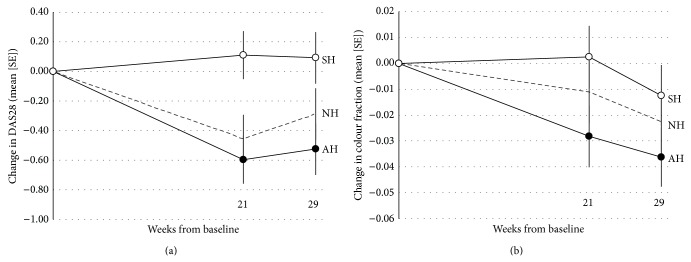
Primary outcome. (a) DAS-28 measures at each time point. (b) Doppler ultrasound measurements. Full line: ○: SH (sham healing). ●: AH (active healing). - - -: NH (no healing). Data are least-squares means (standard errors, mixed linear model estimate) for the intention-to-treat population with the baseline observation carried forward (i.e., conservative).

Changes from baseline in the coprimary outcomes DAS28 and Doppler ultrasound based on the ITT population according to the entire 29-week longitudinal period are illustrated in Figure 2, apparently in favour of active healing (AH) compared to sham healing (SH). After 29 weeks of treatment and follow-up, all outcomes were analysed on the basis of the intention-to-treat population (Table 2). Based on the primary outcome DAS28, there was a statistically significant difference between the groups (ANCOVA, *P* = 0.046). Patients randomised to active healing improved more than the patients allocated to sham healing, corresponding to an average improvement of 0.62 DAS28 points (95% CI: 0.13 to 1.11; *P* = 0.014), which is statistically but not necessarily clinically significant. This improvement translates to an improvement of 17% relative to baseline (3.68). Comparing the patients receiving active healing with those receiving no healing, there was no difference between the groups: 0.24 (−0.25 to 0.73; *P* = 0.34). Although a similar pattern was observed for the mean colour fraction, no statistically significant differences were observed between the groups (ANCOVA, *P* = 0.35).

As the DAS28 is a validated composite index, it was relevant to focus on the individual components included in the calculation of the score. As presented in Table 2, patients randomised to active healing and there was a statistically significant difference between the groups when evaluating tender joint count (ANCOVA, *P* = 0.047), favouring active as opposed to sham healing (2.6, 0.5 to 4.7; *P* = 0.014), corresponding to the improvement in at least two joints. In contrast to this physician-assessed outcome measure, interestingly there was no evidence to support active healing in terms of the patient-reported (ANCOVA, *P* = 0.14) or the physician-reported global health on 100 mm visual analogue scales.

### 3.4. Adherence, Adverse Events, and Patients' Expectations

In the intervention period 0–21 weeks, eleven patients discontinued the study (Figure 1). Most of these patients were excluded due to significant changes in arthritis medication, with more discontinuations for this reason than anticipated in the no healing group. Very few patients withdrew from the study due to lack of interest and none reported adverse events as the cause for withdrawal. Between week 21 and followup at week 29, a further three patients discontinued, all from the active healing group: one patient was lost to followup without any further explanation; one patient did not come to the scheduled visit due to diarrhea and was not given another appointment for logistical reasons; and one patient had diagnosed renal cancer. In retrospect, this patient had a history of microscopic hematuria for a full year prior to entering the study, but he had turned down an offer of further examination with reference to old age. During follow-up, the hematuria worsened to a degree that caused anaemia and the patient finally accepted referral to the oncologist.

Other side effects are as follows: at the visits after treatment and at follow-up, all patients filled in a form about adverse events. Only three patients in the sham healing group reported adverse events of the “healing,” while none did in the active healing group. Neither before nor after therapy did the two groups differ in expectations with respect to the possible effect of healing, and after treatment the participants in the two groups did not differ when asked about the possibility that they had actually received active healing (Table 3).

## 4. Discussion

This study showed a statistically significant, but not clinically, superiority of active healing compared with sham healing with respect to the semiobjective outcome measure DAS28. This change in DAS28 was driven by an objectively measured decrease in the patients' systemic inflammation, with change in favour of AH in joint counts and CRP. In contrast, simply asking the patients to report their (subjective) global health showed no difference between the groups allocated to active and sham healing, indicating a successful blinding. Changes in the Doppler ultrasound measurements did not reach statistical significance.

When planning the study, our primary hypothesis obviously was to compare active and sham healing using an advanced masked design, whereas the third group—those allocated to a treatment-as-usual strategy—was included to enable secondary considerations regarding whether the anticipated subjective benefit in highly motivated patients dedicated to spiritual healing could be explained as a “placebo effect” [19]. The physicians (HB and BDS) and the psychologist (BZ) involved in the study thus anticipated that participating in a study of healing, regardless of being exposed to active or sham healing would induce an effect on the participants. As emphasized by Hróbjartsson and Gøtzsche, when evaluating the placebo effect, a randomised clinical trial needs to compare the placebo (or sham condition as in this case) with a no-treatment condition [19]. Using this approach, the difference found between the double-masked groups cannot be designated as a placebo effect, as the* no healing* group apparently demonstrated a better outcome in terms of both DAS28 and ultrasound than did those receiving sham healing (Figure 2). Although our primary statistical test thus supported the efficacy of active healing, the effect did not correspond to a clinically significant difference. Clinical response, defined as global health improvement ≥50%, was small, with equal prevalence of clinical improvement in the active and sham healing groups (6% of the patients). However, the dual end point, change in ultrasound, and clinical improvement, were not met.

These somewhat puzzling results demand careful consideration of possible bias and potential flaws in the study design. First, the results were based on the relatively robust laboratory values (i.e., CRP), semiobjective joint examinations, and ultrasound evidence of joint inflammation, which were all obtained by independent observers without knowledge of group allocation. The results are therefore based on objective measures rather than subjective patient-reported outcomes (PROs) [20]. Second, the almost identical results of the questionnaire-based outcomes in the active healing and sham healing groups suggest that patients were not consciously disposed to favor or disfavor healing. Third, every effort was made to avoid possible bias, whether positive or negative, from the involved researchers. This was done by blinding any aspect that could be blinded, changing room allocation at random, and dividing responsibility for data handling between several parties. Finally, the responses to the patient-questionnaire concerning whether the patients believed to have received active or sham healing suggested that the attempts to blind patients were successful.

One the aspect of the results that need an explanation are our findings that while active healing differed from sham healing, the improvement in the active healing group did not differ from the no healing, that is, treatment-as-usual, group. The intention in this study was to involve only patients on stable therapy. The anticipated participant was a patient with slight residual arthritis activity that could be managed by means other than changes in systemic medication during the study period. However, as evident from the upper range of the DAS28 at baseline, some participants had highly active arthritis. All these patients belonged to a subgroup not usually seen in the clinic who repeatedly had refused to accept invitations to engage in medical therapy. The no healing group was not blinded, and as indicated by the larger number of protocol violations, that is, patients changing therapy in this group, participants not receiving any healing showed a greater tendency to seek additional therapy, which perhaps could explain the unanticipated improvement seen in this group. Another possible explanation for the observed improvement could be a tendency for “regression towards the mean” generally found in groups of patients volunteering for projects, regardless of group allocation. While participants may have appeared satisfied with their usual therapy at baseline, their reason for joining could have been the fact that they were dissatisfied and hoped for improvement. Finally, even with stratification for prognostic predictors before randomisation, the groups may have differed, resulting in worse outcomes in the sham healing group by chance alone.

## 5. Conclusions

Taken together the results showing that “active healing” differed statistically, not clinically, from “sham healing,” but not from “no healing,” suggest two alternative interpretations. The most conservative explanation would be that the study stumbled upon a group of patients receiving active healing, which by chance experienced a decrease in arthritis activity in comparison with the sham healing group. Chance differences are not uncommon, and the validity of the findings can only be determined by replication of the study. An alternative interpretation is that “energy healing” is in fact able to influence biological processes relevant to rheumatoid arthritis through mechanisms not yet understood by conventional science. While supported by successful efforts to avoid bias through randomisation and blinding, which rules out the possibility that patients receiving healing improved due to effects of expectation or improved psychological coping skills, the latter interpretation is weakened by the results that no healing group showed equally improved outcomes and that the differences generally did not correspond to clinically significant improvement.

In the present well-controlled study, healing was associated with a statistically significant objective outcome in spite of the clinicians' scepticism and the low expectations by the participants. The result is likely to be regarded as a chance of finding by traditional researchers and, in contrast, as an evidence of efficacy, when interpreted by alternatively inclined health providers. The growing interest in CAM from patients and the results from the current study suggest that further well-controlled trials would be relevant to provide evidence for or against the efficacy of spiritual healing.

## Figures and Tables

**Table 1:** Baseline characteristics of the intention-to-treat population.

	Active Healing		Sham Healing		No Healing	
Age (years), mean (SD)	32	60.6 (10.7)	32	58.2 (12.6)	32	58.4 (13.9)
Current/previous use of biologics, number (%)	32	5 (16%)	32	6 (19%)	32	7 (22%)
No use of DMARD^*^/corticosteroid	32	6 (19%)	32	6 (19%)	32	5 (16%)

Disease duration >3 yrs, no. (%)	32	20 (63%)	32	20 (63%)	32	25 (78%)

Disease activity score (28 joints), mean (SD)	32	3.55 (1.28)	32	3.65 (0.94)	32	3.78 (1.64)
Doppler ultrasound (colour fraction: 0-1)	31	0.059 [0.040; 0.198]	30	0.064 [0.029; 0.203]	31	0.076 [0.025; 0.184]

Rheumatoid factor, IU	32	25 [8; 145]	32	27 [10; 227]	32	14 [8; 79]
Erythrocyte sedimentation rate	32	15 [11; 27]	32	16 [11; 23]	32	17 [11; 28]
Tender joint count (28 joints)	32	3 [1; 8]	32	4 [2; 8]	32	2 [1; 10]
Swollen joint count (28 joints)	32	3 [2; 7]	32	4 [3; 6]	32	3 [1; 10]
Patient visual analogue scale (mm)	32	32 [16; 51]	32	45 [19; 62]	32	38 [22; 57]
C-reactive protein concentration (mg/L)	32	2.4 [1.4; 6.7]	32	2.1 [1.0; 5.2]	32	4.2 [0.9; 13.0]
Health Assessment Questionnaire score (0–3)	29	0.40 [0.15; 0.84]	32	0.62 [0.29; 0.95]	31	0.58 [0.16; 0.90]
Physician visual analogue scale (mm)	28	16 [7; 20]	27	15 [7; 23]	29	14 [7; 23]

Values are number of observations (*n*), medians, and interquartile ranges [*Q*1; *Q*3] unless stated otherwise. ^*^Disease modifying antirheumatic drugs.

**Table 2:** Change in outcomes from baseline after 29 weeks.

Outcome variable	Active healing		Sham healing		No healing		ANCOVA^1^
	Mean	(95% CI)	Mean	(95% CI)	Mean	(95% CI)	*P* value
Disease activity score (28 joints)	−0.525	(−0.871; −0.179)	0.092	(−0.254; 0.438)	−0.287	(−0.633; 0.059)	0.046
Doppler ultrasound (colour fraction: 0-1)	−0.036	(−0.059; −0.013)	−0.012	(−0.036; 0.011)	−0.023	(−0.045; 0.000)	0.345

Tender joint count (28 joints)	−2.4	(−3.8; −0.9)	0.2	(−1.2; 1.7)	−1.2	(−2.7; 0.3)	0.047
Swollen joint count (28 joints)	−2.2	(−3.6; −0.8)	0.0	(−1.4; 1.4)	−1.5	(−2.9; −0.1)	0.097
Patient visual analogue scale (mm)	1.8	(−4.9; 8.6)	5.0	(−1.8; 11.7)	−4.4	(−11.1; 2.3)	0.139
C-reactive protein concentration (mg/L)	−3.55	(−6.87; −0.22)	−0.27	(−3.61; 3.08)	−0.58	(−3.90; 2.75)	0.316

Health Assessment Questionnaire score (0–3)	0.0	(−0.1; 0.1)	0.1	(0.0; 0.2)	−0.1	(−0.2; 0.0)	0.051
Physician visual analogue scale (mm)	−3.9	(−8.4; 0.6)	2.6	(−2.0; 7.2)	−2.9	(−7.3; 1.6)	0.101

^1^The analysis of covariance model included the change as the dependent variable, with treatment group being as the main effect and the baseline score as the additional covariate.

**Table 3:** Questions for the participants about subjective feelings regarding healing and CAM in general.

Variable	Active Healing				Sham Healing				AH versus SH
	*N*	Median	*Q*1	*Q*3	*N*	Median	*Q*1	*Q*3	*P*-value
*Baseline *									
Have you tried complementary treatment?^1^	32	88	2	100	32	85	4	96	0.508
Have you tried healing?	32	3	0	46	32	3	0	20	0.800
What are your expectations of participation in this trial?	32	57	46	86	32	47	18	90	0.150
Do you believe complementary treatment is good for arthritis?	32	61	49	80	32	56	32	88	0.265
Do you believe healing is good for arthritis?	32	59	50	73	32	48	22	88	0.184
*After intervention (week 21) *									
How did you feel about participating in this project?^1^	25	100.0	80.0	100.0	24	94.5	58.0	100.0	0.453
Do you think that you were treated by the healer?	25	19.0	0.0	46.0	24	16.0	0.5	47.5	0.880
Do you believe that healing is good for arthritis?	29	50.0	0.0	100.0	27	37.0	0.0	69.0	0.396
Will you use other kinds of complementary treatment?	24	87.5	25.5	100.0	24	47.0	1.0	87.0	0.103
Will you proceed to seek treatment by a healer?	24	34.5	0.0	53.0	23	11.0	0.0	71.0	0.991
*Follow-up (week 29) *									
How did you feel about participating in this project?^1^	27	100.0	66.0	100.0	30	100.0	90.0	100.0	0.422
Do you think that you were treated by the healer?	27	2.0	0.0	46.0	30	15.5	2.0	35.0	0.176
Do you believe that healing is good for arthritis?	27	55.0	7.0	100.0	32	48.5	0.0	94.0	0.363
Will you use other kinds of complementary treatment?	26	99.5	41.0	100.0	30	69.5	17.0	100.0	0.162
Will you proceed to seek treatment by a healer?	23	24.0	0.0	68.0	30	22.0	0.0	62.0	0.841

^1^The answers were given on a 100 mm visual analogue scale on which 0 was defined as “no, not at all” or “fully dissatisfied” and 100 as “yes, definitely” or “fully satisfied.” Values are given as medians with interquartile (*Q*1, *Q*3) ranges. Analysed using Wilcoxon scores: a two-group comparison.

## References

[1] Ernst E. (2000). Complementary and alternative medicine in rheumatology. *Best Practice & Research: Clinical Rheumatology*.

[2] Sleath B., Callahan L. F., Devellis R. F., Beard A. (2008). Arthritis patients' perceptions of rheumatologists' participatory decision-making style and communication about complementary and alternative medicine. *Arthritis & Rheumatology*.

[3] Quandt S. A., Chen H., Grzywacz J. G., Bell R. A., Lang W., Arcury T. A. (2005). Use of complementary and alternative medicine by persons with arthritis: results of the National Health Interview Survey. *Arthritis Care and Research*.

[4] Buchbinder R., Gingold M., Hall S., Cohen M. (2002). Non-prescription complementary treatments used by rheumatoid arthritis patients attending a community-based rheumatology practice. *Internal Medicine Journal*.

[5] Lister J. (1983). Current controversy on alternative medicine. *The New England Journal of Medicine*.

[6] Hodges R. D., Scofield A. M. (1995). Is spiritual healing a valid and effective therapy?. *Journal of the Royal Society of Medicine*.

[7] Benor D. J., Matthews W. J. (2002). Prayer study: what about expectancy effects among the researchers themselves?. *Alternative Therapies in Health and Medicine*.

[8] Zachariae R., Højgaard L., Zachariae C., Væth M., Bang B., Skov L. (2005). The effect of spiritual healing on in vitro tumour cell proliferation and viability—an experimental study. *British Journal of Cancer*.

[9] Arnett F. C., Edworthy S. M., Bloch D. A. (1988). The American Rheumatism Association 1987 revised criteria for the classification of rheumatoid arthritis. *Arthritis and Rheumatism*.

[10] Prevoo M. L. L., Van 'T Hof M. A., Kuper H. H., van Leeuwen M. A., van de Putte L. B. A., van Riel P. L. C. M. (1995). Modified disease activity scores that include twenty-eight-joint counts: development and validation in a prospective longitudinal study of patients with rheumatoid arthritis. *Arthritis and Rheumatism*.

[11] Wells G., Becker J.-C., Teng J. (2009). Validation of the 28-joint disease activity score (DAS28) and European League against rheumatism response criteria based on C-reactive protein against disease progression in patients with rheumatoid arthritis, and comparison with the DAS28 based on erythrocyte sedimentation rate. *Annals of the Rheumatic Diseases*.

[12] Qvistgaard E., Røgind H., Torp-Pedersen S., Terslev L., Danneskiold-Samsøe B., Bliddal H. (2001). Quantitative ultrasonography in rheumatoid arthritis: evaluation of inflammation by Doppler technique. *Annals of the Rheumatic Diseases*.

[13] Altman D. G., Royston P. (2006). The cost of dichotomising continuous variables. *British Medical Journal*.

[14] Ellegaard K., Torp-Pedersen S., Lund H. (2008). Quantification of colour Doppler activity in the wrist in patients with rheumatoid arthritis—the reliability of different methods for image selection and evaluation. *Ultraschall in der Medizin*.

[15] Torp-Pedersen S., Terslev L. (2008). Settings and artefacts relevant in colour/power Doppler ultrasound in rheumatology. *Annals of the Rheumatic Diseases*.

[16] Treasure T., MacRae K. D. (1998). Minimisation: the platinum standard for trials?. *British Medical Journal*.

[17] Altman D. G., Bland J. M. (2005). Treatment allocation by minimisation. *British Medical Journal*.

[18] Cohen J. (1988). *Statistical Power Analysis for the Behavioral Sciences*.

[19] Hróbjartsson A., Gøtzsche P. C. (2010). Placebo interventions for all clinical conditions. *The Cochrane Database of Systematic Reviews*.

[20] Wood L., Egger M., Gluud L. L. (2008). Empirical evidence of bias in treatment effect estimates in controlled trials with different interventions and outcomes: meta-epidemiological study. *British Medical Journal*.

